# Xenon-inhibition of the MscL mechano-sensitive channel and the CopB copper ATPase under different conditions suggests direct effects on these proteins

**DOI:** 10.1371/journal.pone.0198110

**Published:** 2018-06-04

**Authors:** Evgeny Petrov, Gopalakrishnan Menon, Paul R. Rohde, Andrew R. Battle, Boris Martinac, Marc Solioz

**Affiliations:** 1 Laboratory of Biochemistry and Molecular Biology, Tomsk State University, Tomsk, Russia; 2 Victor Chang Cardiac Research Institute, Darlinghurst, Australia; 3 School of Biomedical Sciences, Queensland University of Technology (QUT), Brisbane, Australia; 4 St Vincent’s Clinical School, University of New South Wales, Darlinghurst, Australia; 5 Department Clinical Research, University of Bern, Bern, Switzerland; University of Technology Sydney, AUSTRALIA

## Abstract

Xenon is frequently used as a general anesthetic in humans, but the mechanism remains an issue of debate. While for some membrane proteins, a direct interaction of xenon with the protein has been shown to be the inhibitory mechanism, other membrane protein functions could be affected by changes of membrane properties due to partitioning of the gas into the lipid bilayer. Here, the effect of xenon on a mechanosensitive ion channel and a copper ion-translocating ATPase was compared under different conditions. Xenon inhibited spontaneous gating of the *Escherichia coli* mechano-sensitive mutant channel MscL-G22E, as shown by patch-clamp recording techniques. Under high hydrostatic pressure, MscL-inhibition was reversed. Similarly, the activity of the *Enterococcus hirae* CopB copper ATPase, reconstituted into proteoliposomes, was inhibited by xenon. However, the CopB ATPase activity was also inhibited by xenon when CopB was in a solubilized state. These findings suggest that xenon acts by directly interacting with these proteins, rather than *via* indirect effects by altering membrane properties. Also, inhibition of copper transport may be a novel effect of xenon that contributes to anesthesia.

## Introduction

There has been substantial interest in using noble gases as 'ideal' general anesthetics for more than half a century [1,2]. Noble gases are also useful as a tool in biophysical studies of properties of biological membranes and their protein constituents. Being chemically inert, noble gases have preferential affinity for the hydrophobic environment, which provides an opportunity to directly manipulate the functioning of phospholipid membranes [3–5]. The effectiveness of volatile anesthetics follows the Meyer-Overton rule [6,7], which states that a greater solubility in olive oil is correlated to a greater anesthetic potency [8]. This describes, but does not explain the anesthetic effects. It has remained a matter of debate whether volatile anesthetics solely work by nonspecific effects on the membrane, or also *via* a specific interaction with membrane receptors, pumps, or channels.

As xenon is highly soluble in the lipid bilayer, its presence will affect the properties of the bilayer. In particular, when dissolved in phospholipid bilayers, xenon influences parameters such as membrane thickness [5], lateral pressure profile [9], melting point and phase transition temperature [4]. By altering membrane properties, xenon may affect the function of integral membrane proteins indirectly. On the other hand, it was shown that xenon also binds to hydrophobic sites within proteins. This was demonstrated for both, soluble proteins and integral membrane proteins. X-ray diffraction studies of a number of enzymes have revealed that xenon binds to hydrophobic cavities in proteins through weak but specific dipole-induced dipole interactions [10]. In the membrane-embedded cytochrome *ba*_3_ oxidase of *Thermus thermophilus*, xenon atoms were shown to line the hydrophobic channel that provides access of O_2_ to the *a*_3_-Cu_B_ center [11].

The GABAergic system has been considered as a prime target for mediating the anesthetic effects of xenon [12–14]. More recently, xenon was shown to competitively inhibit the *N-*methyl-D-aspartate receptor by binding to the glycine binding site [15][16]. Xenon also acts on other channels like TREK-1 or adenosine triphosphate-sensitive potassium (K_ATP_) channels and several of these inhibitory effects contribute to the neuroprotection against traumatic brain injury by xenon [17–19]. Detailed mapping of the xenon binding sites by X-ray crystallography was conducted on the ligand-gated ion channel GLIC of *Gloebacter violaceus* [20]. GLIC is a member of the pLGIC family, which also includes serotonin, n-acetylcholine, glycine, and GABA receptors. Xenon binding to three distinct regions in the trans-membrane domain of GLIC was observed: to an intra-subunit cavity, at the interface between adjacent subunits, and in the pore, which was effectively sealed by the bound xenon. Clearly, intra-protein binding of xenon is a pharmacologically relevant mechanism, although this does not rule out the possibility that xenon directly affects membrane properties, which can in turn affect the function of integral membrane proteins within.

To compare the effects of xenon on a protein under different membrane conditions, we measured xenon-inhibition of the mechano-sensitive channel of large conductance (MscL) of *Escherichia coli* at ambient and high pressure and the copper transporting ATPase CopB of *Enterococcus hirae* in solution and in a membrane-bound state. MscL is a robust mechano-sensitive channel for multiple ions (non-selective) and its gating activity can be modulated by physical [21–23] or chemical [24–28] means. MscL of *E*. *coli* is homologous to MscL of *Mycobacterium tuberculosis*, for which an X-ray structure is available [29]. The crystal structure shows that the channel is a homo-pentamer. Each monomer contains two transmembrane helices and has the N- and C-terminal domains located on the cytoplasmic side of the membrane. The first of the two transmembrane domains that exist per subunit contain a cluster of hydrophobic residues which form a ‘hydrophobic lock’ within the pentameric channel pore. Membrane thickness and hydrophobic mismatch between the MscL protein and the membrane have a direct impact on the functional state of wild-type MscL [21,23,25,26]. In this study, we used a G22E mutant of *E*. *coli* MscL (MscL-G22E) to evaluate the effects of xenon on the channels gating activity. The G22E mutation induces the channel to spontaneously open with a gating function without a tension being deliberately applied to the membrane. It was found that xenon completely inhibits the spontaneous activity of MscL-G22E. This effect could be reversed by applying increased hydrostatic pressure to membrane patches containing these channels, suggesting direct effects of xenon on the protein.

To obtain further evidence for direct effects of xenon on a membrane protein, we used the CopB copper ATPase of *E*. *hirae* [30]. The copper pumping activity by CopB is directly linked to ATP hydrolysis: if no copper ions are available for transport, no ATP hydrolysis takes place [31]. This allows the recording of CopB activity not only in a membrane environment, but also in a solubilized state in the absence of phospholipids by monitoring ATP hydrolysis. Xe inhibited CopB in both, the membrane-bound and the solubilized state. These observations suggest that xenon primarily exerts its inhibitory effect directly on the membrane proteins studies here, rather than *via* changes of membrane properties.

## Materials and methods

### Cell-free expression construct and MscL-G22E

Wild-type MscL was PCR-amplified from the MscL pGEX1.1 expression vector [32], employing primers 5'-gacctgcagctggttccgcgtgga and 5'-attaagctttcaggcgcttgtta, followed by subcloning into pQE-31 (Qiagen, Germantown, MD), cut with PstI and HindIII. In the resulting clone, *MscL 2*.*1*, MscL is endowed with a thrombin-cleavable N-terminal histidine tag, resulting in the following amino acid sequence before MscL’s Met1: MRGSHHHHHHTDPHASSVPRVDLQLVPRGSLEHRENN. The N-terminus after thrombin cleavage is underlined. For cell-free expression using *E*. *coli* extracts, the coding sequence was subcloned by PCR with primers 5’-AAATTTCATATGAGAGGATCTCACCAT and 5’-TAGGTAAAGCTTTCAGGCGCTTGT into the NdeI and HindIII sites of pET-17b (Novagen), resulting in clone *MscL 3*.*1*. The MscL-G22E mutation was obtained by site-directed mutagenesis of clone MscL 2.1 with the QuikChange II kit (Stratagene, La Jolla, CA), using primers 5'-gtggatttggcggtggaggtcattatcggtgcgg and 5'-ccgcaccgataatgacctccaccgccaaatccac.

MscL-G22E was produced using a cell-free expression system essentially as described previously [33]. Briefly, the inner reaction was scaled to 2.52 mL with 30 mL outer buffer, and incubated for 13 h with longitudinal shaking at 200 min^-1^ and a ¾-inch stroke at 37 °C. The inner reactions were centrifuged for 20 min at 12,000 x g at 4 °C. An 8 μL aliquot of each supernatant was run on a 12% SDS-polyacrylamide gel for analysis. MscL-G22E was solubilized with 1% *n*-dodecyl-β-D-maltoside (Affymetrix, Santa Clara, CA) and combined with 450 μL of PBS-washed Talon metal affinity resin (Clontech, Mountain View, CA) to allow binding to the 6His-MscL-G22E for 2 h. The resin was then pelleted and washed with 1 mM *n*-dodecyl-β-D-maltoside in PBS pH 7.5, and re-suspended to a final volume of 100 μL in the same buffer. MscL-G22E was released from the affinity resin by cleavage with 0.58 U of thrombin (Biopur, Bubendorf, Switzerland) at 4 °C for three days. MscL released from the column was analyzed by SDS gel electrophoresis (Figure A in S1 File).

### Liposome reconstitution of MscL-G22E

Purified MscL-G22E was reconstituted into soybean phospholipid liposomes using a dehydration/rehydration (D/R) technique, modified from Häse *et al*. [32] and Delcour *et al*. [34]: 20 mg of dry soybean phospholipids were placed in a glass centrifuge tube and dissolved in 1–2 mL of chloroform. The chloroform was evaporated with a stream of nitrogen with constant rotating of the tube to form a uniform lipid layer without lumps, followed by 20 min of drying with a stronger N_2_ stream. The dried soybean phospholipids were resuspended in 2 mL of D/R buffer (200 mM KCl, 5mM K-HEPES, pH 7.2) using a small brush, followed by sonication for 10 min in a bath sonicator to obtain a transparent, opalescent suspension. MscL-G22E was mixed with 200 μL of lipid suspension to achieve a protein/lipid mass ratio of 1:1000. The mixture was diluted with D/R buffer to 3 mL and mixed on a platform rocker for 1 h, then 20–30 mg of Biobeads (BioRad, Hercules, CA) were added for a further 3 h. The beads were allowed to settle, and the supernatant was centrifuged at 440,000 x g for 30 min. The pellet was resuspended in 40 μL D/R buffer and 20 μL aliquots were put as spots on microscope cover glasses and dehydrated overnight in a desiccator. The spots were then rehydrated by placing 15–20 μL of D/R buffer on each spot and left for 24 h at 4 °C. A small amount (1–2 μL) of proteoliposomes was placed in the recording chamber containing the recording solution. Thin-walled bubbles started to appear on the sides of lumps of lipid after 10–20 min and were used for patch-clamping.

### Patch clamp and “flying-patch” electrophysiology of MscL-G22E

Spontaneous activity of MscL-G22E was recorded using the patch clamp technique in voltage clamp mode [35]. For measurements at increased hydrostatic pressure, the “flying-patch” methodology previously described was used; it is chiefly a mechanical device that allows easy transfer a membrane patch to high-pressure conditions [36,37]. If MscL-G22E was active for at least 5 min, recording and data analysis were conducted as previously described [36,37]. All recordings were repeated 3 to 7 times.

### Measurements with xenon

To saturate buffer with xenon, a 500-ml bottle containing buffer was attached to a cyclic saturator (Figure B in S1 File) and xenon was pumped through the solution at 4 °C overnight. Xenon-saturated solutions were stored in 5 mL aliquots in sealed syringes at -80 °C. The effectiveness of Xe-saturation of solutions was measured by mass spectrometry with a DSQ II Dual Stage Quadrupole GC/MS spectrometer, using the ^132^Xe isotope and calibration with gas samples containing 5%, 15% and 100% of xenon in air at atmospheric pressure. Based on the calibration curves (Figure C in S1 File), the xenon-saturated buffers contained ~6% (v/v) Xe. To determine the effects of xenon on protein activities, xenon-saturated buffer was added to patch clamp recordings or was used as assay buffer to measure ATPase activities. Since xenon diffuses only very slowly out of solution, the assays could be performed without any further precautions.

### CopB ATPase purification

The *E*. *hirae* mutant Yl that lacks the CopY repressor and thus overexpresses the *cop* operon about 50-fold was used for CopB purification [38]. Y1 cells were grown in semi anaerobic conditions in two liters of N-media (60 mM Na_2_HPO_4_, 1% peptone, 0.5% yeast extract, 1% glucose, pH 7.8–8.0) at 37 °C to an OD at 600 nm of 1 to 1.5 and centrifuged at room temperature for 12 min at 8000 x g. The cells were washed in 1/10 volume of 2 mM MgSO_4_ by centrifuging at room temperature for 12 min at 8000 x g. The wet weight of the cells was determined and cells were resuspended in 6 mL/g wet cells of 250 mM K_2_SO_4_, 2 mM MgSO_4_, 50 mM K-HEPES, pH 7.2. Lysozyme (4 mg/g wet cells) was added and the suspension incubated in a shaking water bath at 37 °C for 45 min. Then, 200 μg/g of wet cells of DNase 1 was added and incubation continued for 15 min. The cells were centrifuged for 12 min at 23,000 x g and 4 °C and resuspended in 2 ml/g wet cells of 20 mM Tris-SO_4_ pH 7.5, 5 mM MgSO_4_, 25 mM Na_2_SO_4_, 25 mM K_2_SO_4_, 1 mM β-mercaptoethanol, 1 μM CuSO_4_, 20% (v/v) glycerol) and 1/100 volume of protease inhibitor cocktail (Table A in S1 File) and the pellet re-suspended using a Potter-Elvehjem homogenizer at 1000 rpm. Cells were then lysed by passing the suspension twice through a French press at 20 MPa. Cells and membranes were collected by centrifugation for 60 min at 90000 x g at 4 °C. The membrane pellet was resuspended in 0.66 mL of buffer G per g wet of cells and frozen at -20 °C. The membrane protein concentration was determined with the Bradford assay [39], using bovine serum albumin as the standard. Frozen membrane suspensions were stable for 3 months.

For CopB extraction, frozen membrane suspensions were thawed, 1/200 volume of protease inhibitor cocktail (Table A in S1 File) and 1 g of dodecyl-β-D-maltoside were added and the suspension was stirred on ice for 1 h. Extracted membranes were removed by centrifugation for 45 min at 90,000 x g and 4 °C. All further steps were conducted at 4 °C. The supernatant was supplemented with 10 mM imidazole-SO_4_ pH 7.5 and loaded onto a Ni-NTA-column, pre-equilibrated with JD-buffer [20 mM Tris-SO_4_ pH 7.5, 5 mM MgSO_4_, 1 mM β-mercaptoethanol, 1 μM CuSO_4_, 20% glycerol, 0.05% dodecyl-β-D-maltoside (DM)]. The column was washed with JD-buffer containing 10 mM imidazole-SO_4_ pH 7.5, followed by JD-buffer containing 50 mM imidazole-SO_4_ pH 7.5. CopB was eluted with JD-buffer containing 200 mM imidazole-SO_4_ pH 7.5. The fractions were analyzed by 10% SDS-PAGE [40] and protein bands were visualized by staining with colloidal Coomassie blue (Figure D in S1 File). The fractions containing CopB were dialyzed against 250 ml of buffer A (20 mM TrisSO_4_, pH 7.5) containing 0.05% DM for 2.5 h, followed by freezing at -80 °C.

### Reconstitution of CopB

To reconstitute CopB into lipid vesicles, 10 mg of soybean phospholipids dissolved in ether were dried with a stream of N_2_, followed high vacuum-drying for 10 min. The dried lipids were suspended in 800 μl of buffer A containing 20 mg of octyl-β-D-glucoside. The clear lipid micellar solution was equilibrated on ice for at least 10 min. All further steps were carried out at 4 °C. To the lipid solution, 200 μg of purified CopB were added, immediately followed by dialysis against 100 ml of buffer A for 2.5 h and buffer A containing 1 mM DTT for 14 h.

### ATPase assays

ATPase activity of purified CopB as well as reconstituted CopB was measured by determining the release of Pi by the method of Lanzetta [41]. The 1 ml reaction mixtures for the solubilized CopB consisted of air-saturated or xenon-saturated 50 mM Na-MES, pH 6.5, 0.1% DM, 2.5 mM DTT, 5 mM MgSO_4_, 1 μM CuSO_4_, 1 mg/mL soybean phospholipids, and 3.75 μg of CopB. The reaction was conducted at 37 °C and started by the addition of 1 mM of Na-ATP pH 7. At 0, 5, 10, 15, and 20 min, 100 μL aliquots were removed and Pi determined as described [41]. The ATPase activity of reconstituted vesicles was determined similarly, except that the 1 ml reactions consisted of vesicles containing 7.15 μg of CopB, 50 mM Na-MES, pH 6.5, 2.5 mM DTT, 5 mM MgSO_4_, 1 μM CuSO_4_ and 20% (v/v) glycerol. Specific activities were calculated from the slopes of linear regressions (n = 3).

## Results

### Effect of xenon on MscL-G22E

**Fig 1:**
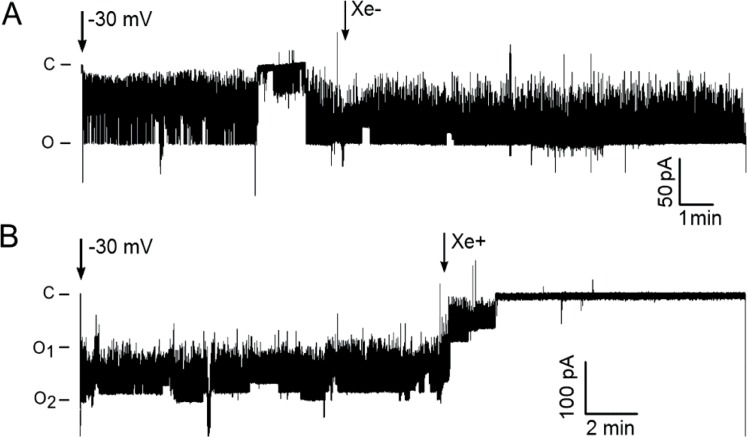
Effect of xenon on MscL-G22E spontaneous activity. A. The current induced by MscL-G22E was recorded at a potential of -30 V in Xe-free buffer. At the arrow, 1/3 volume of xenon-free (Xe-) recording solution was added. B. As in A, but at the arrow, xenon-saturated (Xe+) recording solution was added. The ordinates indicate the currents corresponding to the closed state (c) and open states (O_1_, O_2_) of at least two spontaneously active channels.

The choice to utilize the gain-of-function mutant MscL-G22E instead of the wild-type MscL was based on the observation that MscL-G22E is spontaneously active in non-stretched membranes while retaining its mechanosensitivity, resulting in a greater open probability [42]. To exclude side-effects of mechanical disturbance due to increased levels of liquid in the recording chamber and shaking of the membrane patch, control recordings with xenon-free solution were performed. As seen in Fig 1A, channel activity remained relatively constant over several min. Addition of 0.5 ml of Xe-free recording bath solution did not fundamentally change the gating activity of the channels. After addition of 0.5 mL of Xe-saturated bath solution to the recording chamber, MscL-G22E became inhibited within 5 ± 2 min (n = 7) (Fig 1B). This is seen as both a significant decrease in open probability of an ensemble of two spontaneously active channels (O1, O2) and by suppression of flickering activity.

**Fig 2:**
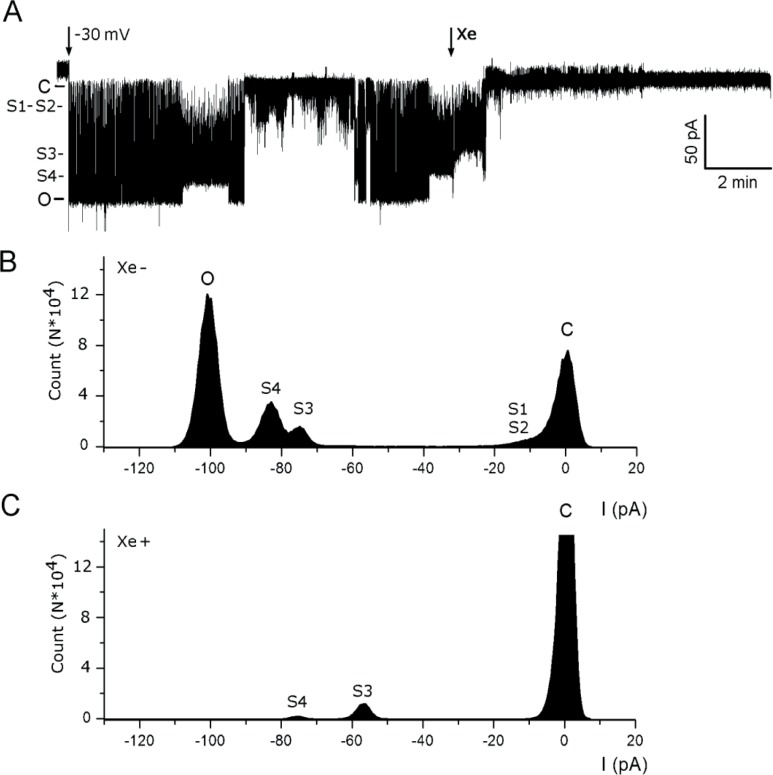
Effect of xenon on MscL-G22E activity and sub-state distribution. A. The current induced by a single MscL-G22E channel was recorded at a potential of -30 V in Xe-free buffer. At the arrow, 1/3 recording chamber volume of xenon-saturated (Xe) recording solution was added. B. Frequency distribution of conductance events due to open states (O), open sub-states (S1, S2, S3, S4) and closed states (C) in the absence of xenon. C. As in B, but in the presence of xenon.

We also observed that the fine-structure of gating by MscL-G22E was affected by the presence of xenon. To visualize these subtle changes in sub-states of the channel, it was necessary to obtain patches containing only one or two channels. Under these conditions, it became apparent that in addition to the open and closed states, there are intermediate open states S1, S2, S3 and S4 (Fig 2). Xenon abolished all open states of MscL-G22E and reduced the activity of the channel to a very low flickering activity near the closed state.

**Fig 3:**
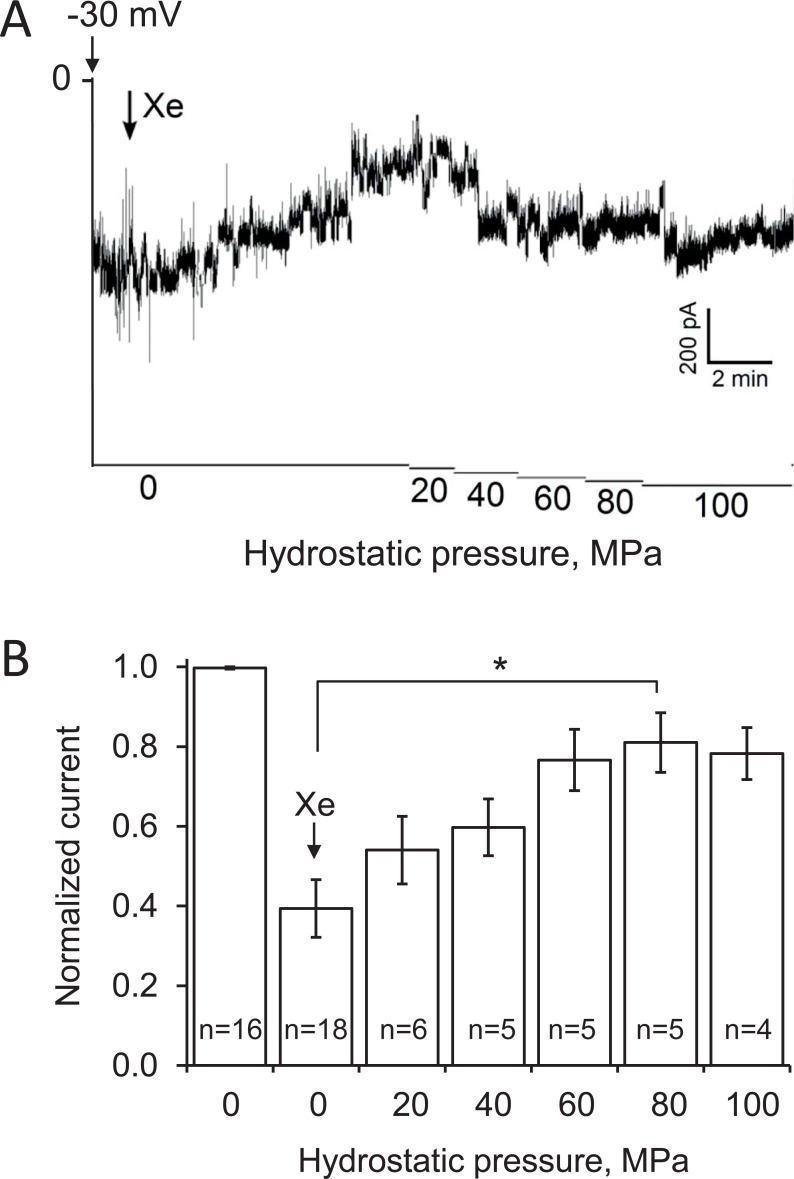
Reactivation of xenon-inhibited MscL-G22E by hydrostatic pressure. A. Patch-clamp recording with a membrane patch with an ensemble of >10 channels. Voltage and xenon applications are marked with arrows. The hydrostatic pressure was increased stepwise as indicated by the abscissa. B. Quantitative evaluation of the effects of xenon and hydrostatic pressure on MscL-G22E spontaneous activity. The data were collected from recordings like that shown in A. The error bars indicate the standard error; the number of evaluated recordings, n, is indicated in the bars. The asterisk indicates a significant difference of p ≤ 0.01.

While the inhibition of the MscL-G22E channel by xenon is clearly apparent, it remains unclear if this is due to changes of the membrane properties or interaction of xenon with hydrophobic domains or pockets of the protein. To address this question, we examined the effect of xenon on the MscL-G22E channel under increased hydrostatic pressure, which has previously been shown to increase the open-probability of this channel [37]. Fig 3 shows that upon xenon exposure, MscL-G22E channel activity was reduced to 39 ± 7% (p < 0.0001). A step-wise increase in hydrostatic pressure reactivated the channels, reaching maximal recovery at 80 MPa (81 ± 7%, p = 0.01, Fig 3B). Higher hydrostatic pressure did not lead to significantly higher recovery of activity. Higher hydrostatic pressure, which also results in an increased xenon partial pressure, *p*_Xe_, leads to additional thickening of the membrane [4]. If xenon would act *via* changes of membrane properties, elevating the hydrostatic pressure would be expected to induce channel closing, rather than the opening observed here. How pressure reactivates the xenon-inhibited channel remains speculative. It has been proposed that under pressure, water molecules around the hydrophobic gate of MscL-G22E induce hydration of the hydrophobic lock, thus increasing the opening probability of the channel [37]. This could lead to a reversal or overriding of xenon inhibition. Whatever the mechanism of high-pressure reversal of xenon inhibition is, it supports a direct action of xenon on MscL-G22E, rather than indirect effects *via* the membrane as the primary inhibitory mechanism.

To strengthen the concept that xenon inhibition can results from direct interaction of xenon with the protein, rather than the membrane, we also assessed the effects of xenon on the CopB copper ATPase. The activity of this enzyme can be measured in solution as well as in the membrane bound state.

### Effects of xenon on solubilized CopB

The ATPase activity of the CopB copper ATPase is tightly linked to copper transport, and thus serves as a measure of transport activity that can effectively be determined on the solubilized enzyme as well as on the enzyme reconstituted into membrane vesicles [31]. Since CopB naturally possesses a very histidine-rich N-terminal domain, it can be purified by Ni-NTA affinity chromatography without a need to modify the protein [43]. It could thus be purified from its native host as a protein which is at least 90% pure (Figure D in S1 File).

**Fig 4:**
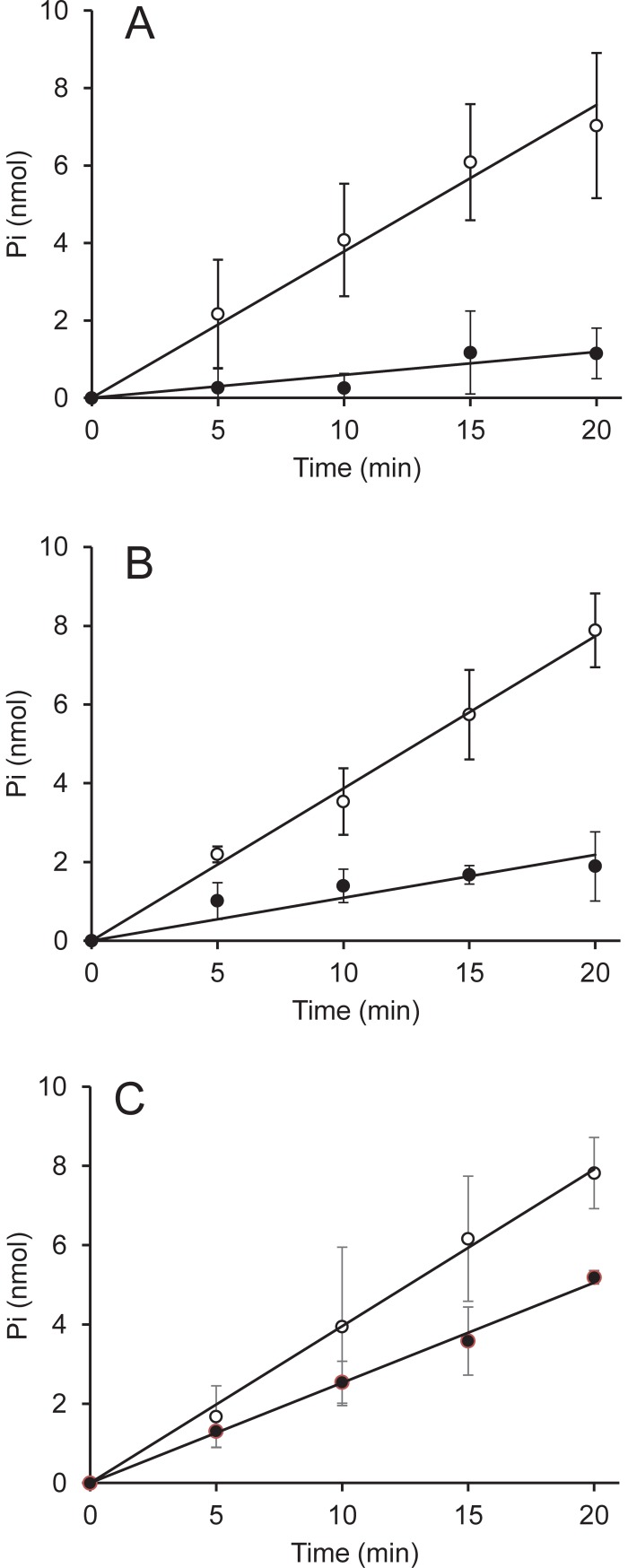
ATPase activity of CopB was measured either without (○) or with (●) xenon in the buffer. A. Solubilized CopB in the presence of 0.1% dodecylmaltoside. B. Solubilized CopB in the presence of 0.1% dodecylmaltoside and 10 μg/ml of soybean phospholipids. C. Reconstituted CopB in proteoliposomes. Each data point represents the release of Pi by 0.337 μg (A, B) or 0.715 μg (C) of CopB. Other details of the measurements were conducted as detailed under Material and methods. The error bars indicate the standard deviation of three independent experiments (n = 3).

Solubilized CopB will have a comparatively small annulus of detergent surrounding the hydrophobic domain, but not the large hydrophilic portion of the protein harboring the ATP-binding and -hydrolyzing domains. Xenon-effects observed on lipid bilayers are thus unlikely to be mimicked by the small detergent annulus. When the activity of solubilized, purified CopB was measured in the absence of membrane lipids, it amounted to 976 ± 215 nmol min^-1^ mg^-1^ (Fig 4A, Table 1). Xenon inhibited this activity by 85%. Since no membrane was present in this experiment, the observed inhibition suggests that xenon affected the enzyme by directly targeting the protein.

**Table 1:** Specific activity and xenon-inhibition of different CopB preparations.

CopB preparation	nmol min^-1^ mg^-1^^a^	%^a^	Significance^b^
**Soluble**	976 ± 215	100	0.008
**Soluble+Xe**	155 ± 87	15.0	
**Soluble+phospholipids**	1046 ± 121	100	0.0007
**Soluble+phospholipids+Xe**	266 ± 109	24.9	
**Resonstituted**	541 ± 81	100	0.0008
**Reconstituted+Xe**	339 ± 68	62.4	

^a^Values with standard deviation (n = 3).

^b^Significance p of the difference was calculated with Student's t-test (n = 3).

The addition of phospholipids to solubilized CopB will result in a mixed detergent-phospholipid annulus covering the hydrophobic domain of the protein. The basal activity of CopB under these conditions was 1046 ± 121 nmol min^-1^ mg^-1^ and thus not significantly different from that in the absence of phospholipids (Fig 4B, Table 1). In the presence of phospholipids, xenon inhibited CopB somewhat less, namely 75%, *versus* 85% in the absence of phospholipids (p < 0.001). However, if xenon were to inhibit CopB *via* altered phospholipid properties, *higher* rather than lower inhibition of CopB would be expected in the presence of phospholipids, Overall, the xenon-inhibition of CopB not integrated into a lipid bilayer suggests direct xenon-protein interaction.

### Effects of xenon on reconstituted CopB

Purified CopB reconstituted into proteoliposomes by the method described here resulted in a relatively homogeneous population of unilamellar vesicles of 50 to 100 nm diameter (Figure E in S1 File). Reconstituted CopB exhibited an ATPase activity of 541 ± 81 nmol min^-1^ mg^-1^ (Fig 4C and Table 1). The lower activity of this preparation compared to soluble CopB is due to the random orientation of CopB in the membrane: only molecules with the cytoplasmic, ATP-binding domain, facing outside of the vesicles can be activated by externally added ATP. The activity of membrane-bound CopB was reduced to approximately 62% by xenon.

Clearly, the liposome membrane reduced xenon-inhibition of reconstituted CopB. However, the fact that solubilized CopB is more strongly inhibited by xenon than reconstituted CopB suggests that the membrane exerts a *stabilizing* effect on CopB and partly suppresses xenon inhibition. If membrane effects would inhibit CopB, more extensively, stronger inhibition of reconstituted CopB by xenon should have been observed. Thus, the reduced xenon-inhibition of reconstituted CopB corroborates a mechanism of xenon inhibition by direct protein interaction. Inhibition of an ion-motive ATPase by xenon has not previously been demonstrated and could have far-reaching implications in terms of general anesthesia.

## Discussion

Mechanosensitive channels act as membrane-embedded mechano-electrical switches by opening an ion-conducting pore in response to lipid bilayer deformations. This process is a key to physiological responses of living organisms to mechanical stimuli, such as in touch, hearing, or osmoregulation. The osmo-regulatory protein MscL of *E*. *coli* changes its diameter from 50 Å to 70 Å when transitioning from the fully closed to the fully open state [44]. This nearly two-fold increase of the area occupied by MscL in the membrane explains why MscL readily responds to membrane lateral pressure and is thus a mechano-sensitive channel. In this study, we show for the first time that MscL-G22E, a spontaneously active mutant of MscL, is inhibited by xenon. Xenon inhibition of a membrane protein can come about by two fundamentally different mechanisms: by direct interaction of xenon with the protein, or by changes of the membrane properties as a consequence of the partitioning of xenon into the membrane.

Xenon readily diffuses into the hydrophobic region of phospholipid bilayers where it forms clathrates. Modelling studies have shown that Xe atoms are mostly located between the leaflets of the bilayer [4,5]. Xe binding to the membrane is accompanied by thickening of the membrane and a shift in the phase transition temperature [3][45]. Hydrophobic interactions are crucial for mechano-sensitive channel gating [23] and changes in membrane properties, such as the phase transition temperature, viscosity or thickness could well be the explanation of the xenon-inhibition of MscL-G22E function. In a reconstituted system where the lipid to protein ratio is typically in excess of 50 and membrane structural proteins are absent, no lateral membrane pressure can build up, even in the presence of xenon. But xenon partitioning into the membrane leads to membrane thickening, which in principle could inhibit MscL by hydrophobic mismatch between the bilayer and the channel protein [23]. Increased hydrostatic pressure enhances membrane thickening and would thus be expected to result in stronger inhibition of the channel protein. However, the opposite was observed: increased hydrostatic pressure *relived* inhibition of MscL-G22E by xenon, which supports a model of xenon-inhibition of MscL-G22E by interaction with the protein. Clearly, considerable additional work will be required to elucidate such a mechanism, but it will be important to investigate along these lines.

The activity of the CopB copper-pumping ATPase can be assessed both in the membrane-bound and in the solubilized state. The pumping of ions by CopB is directly coupled to ATP hydrolysis: if pumping is inhibited or the ions to be pumped are absent, no ATP hydrolysis takes place [46]. Since xenon inhibits the ATPase activity of CopB to the same extent in the membrane-bound and the solubilized state, the gas most likely acts by direct binding to the protein. While this mechanism of inhibition of CopB by xenon requires further verification, xenon-inhibition of an ion-pumping ATPase by xenon *per se* is novel and has far-reaching ramifications. Eukaryotic cell function depends on the operation of membranous ion pumps, like the Na,K-ATPase, the Ca-ATPase, or copper ATPases. The two human copper ATPases, ATP7A and ATP7B are closely related to CopB and other bacterial copper pumps [47] and are inextricably linked to satisfying systemic and central nervous system requirements for copper [48]. Copper also plays a key role at the synapse and in synaptic transmission and in the past decade, a physiological role for copper in regulating neuronal excitability has emerged [49]. Xenon-inhibition of neuronal copper ATPases would of course have far-reaching consequences on brain function and may contribute or even be central to the anesthetic effects of this noble gas. None of our findings precludes the possibility that in addition to direct action of xenon on proteins, xenon can affect channel and pump functions by membrane-exerted mechanisms caused by xenon accumulation in the lipid bilayer.

## Conclusion

Xenon is used as an anesthetic in human medicine and consequently, there is great interest in its molecular mode of action. Since xenon is hydrophobic, it readily partitions into membranes as well as hydrophobic cavities in proteins. Since there are multiple targets for xenon, the mechanism of the anesthetic effects of the gas has been difficult to resolve. It is generally assumed that the anesthetic effect is due to inhibition of one or several gated neuronal channels. As a proof of concept we here show that the spontaneously gating MscL-G22E mutant of the *E*. *coli* mechano-sensitive MscL channel is also inhibited by xenon. As a second model system for an integral membrane protein, the CopB copper ATPase of *E*. *hirae* was used. Its activity can be assessed both, in a membrane-embedded state and in solution. Xenon inhibits CopB when reconstituted in proteoliposome or when in solution, suggesting that inhibition is governed by direct interaction of the gas with the protein. Since copper ATPases play a pivotal role in brain function, the inhibition of copper pumps by xenon points to a novel possible mode of action of xenon in anesthesia.

## Supporting information

S1 File All Supporting information. (Figure A) Purification of MscL-G22E. (Figure B) Device for saturation of solution with xenon. (Table A) Protease inhibitors and composition of 100x stock solution. (Figure C) Determination of the xenon concentration in buffers. (Figure D) Purification of CopB. (Figure E) Freeze-fracture electron micrographs of CopB proteoliposomes.
(PDF)
