# Supplementary material for: Xenon-inhibition of the MscL mechano-sensitive channel and the CopB copper ATPase under different conditions suggests direct effects on these proteins
Source: PLoS One. 2018 Jun 4;13(6):e0198110. doi: 10.1371/journal.pone.0198110 (PMC5986136; doi:10.1371/journal.pone.0198110)
Supplement: S1 File — (Figure A) Purification of MscL-G22E. (Figure B) Device for saturation of solution with xenon. (Table A) Protease inhibitors and composition of 100x stock solution. (Figure C) Determination of the xenon concentration in buffers. (Figure D) Purification of CopB. (Figure E) Freeze-fracture electron micrographs of CopB proteoliposomes. (PDF) [file pone.0198110.s001.pdf]

Supporting information

Xenon-inhibition of the MscL mechano-sensitive channel and the CopB copper ATPase under different conditions suggests direct effects on these proteins

Evgeny Petrov[1¶], Gopalakrishnan Menon[1¶], Paul R. Rohde[2], Andrew R. Battle[2,3], Boris Martinac[2,4], Marc Solioz[1,5]

[1]Laboratory of Biochemistry and Molecular Biology, Tomsk State University, Tomsk, Russia

[2]Victor Chang Cardiac Research Institute, Darlinghurst, Australia

[3]School of Biomedical Sciences, Queensland University of Technology (QUT), Brisbane, Australia

[4]St Vincent's Clinical School, University of New South Wales, Darlinghurst, Australia

[5]Department Clinical Research, University of Bern, Bern, Switzerland

*Corresponding author

E-mail: marc@solioz-scientific.ch

[¶]These authors contributed equally to this work.

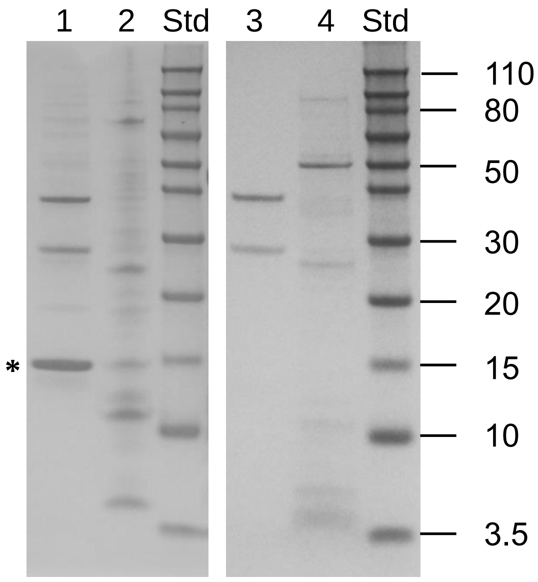

Figure A. Purification of MscL-G22E. Lane 1, approximately 2 μg of MscL-G22E released from the column by 0.58 U of thrombin; lane 2, total fraction of plasmid-directed *in vitro* protein synthesis bound to the column; lane 3, 0.58 U of thrombin used for cleavage; lane 4, unrelated sample; Std, molecular weight standards as indicated in kDa in the Figure. The Figure shows 12% polyacrylamide/SDS gels stained with SimplyBlue SafeStain (Novex); the asterisk indicates MscL-G22E.

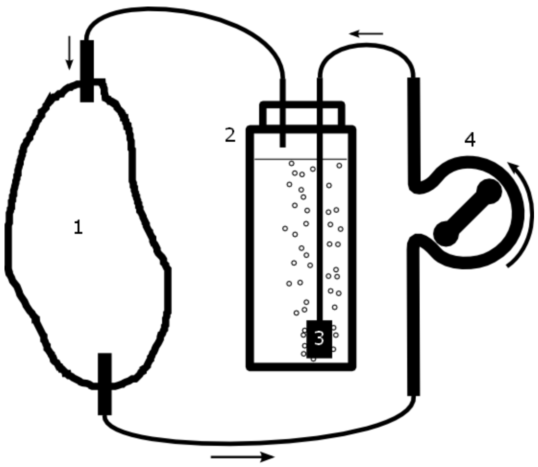

Figure B. Device for saturation of solution with xenon. 1, Plastic bag with 2 l of xenon; 2, glass jar (500 ml) with recording solution; 3, gas diffuser; 4, peristaltic pump.

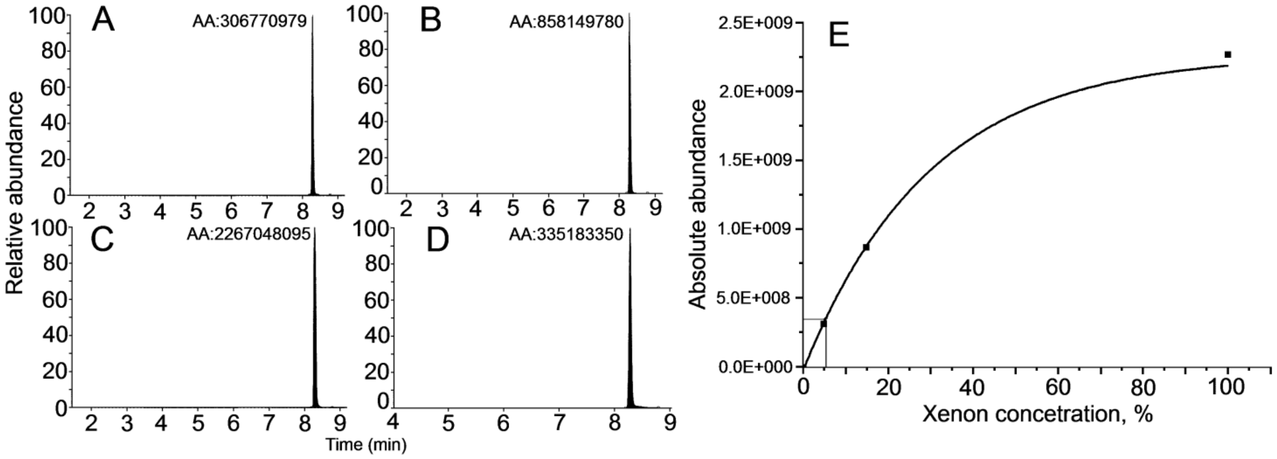

Figure C. **Determination of the xenon concentration in buffers.** Mass spectra of calibration solutions containing 5% (A), 15% (B), and 100% (C) xenon in air, and of a saturated buffer solutions (D). E. Calibration curve for xenon measurements. AA, absolute abundance of ^{132}Xe.

Table A. Protease inhibitors and composition of 100x stock solution

**Abbreviation**	**Chemical name**	**Source**	**M_r**	**Per 5 ml DMSO[1]**
PMSF	Phenylmethylsulfonylfluoride	Merck 7349	174	87 mg
TLCK	N-Tosyl-L-lysylchloromethane	Merck 24648	369	184 mg
TPCK	N-Tosyl-L-phenylalanyl chloromethane	Merck 8615	352	176 mg
pABA	*p*-Aminobenzamidine	Sigma A7148	208	104 mg

[1]DMSO, dimethylsulfoxide

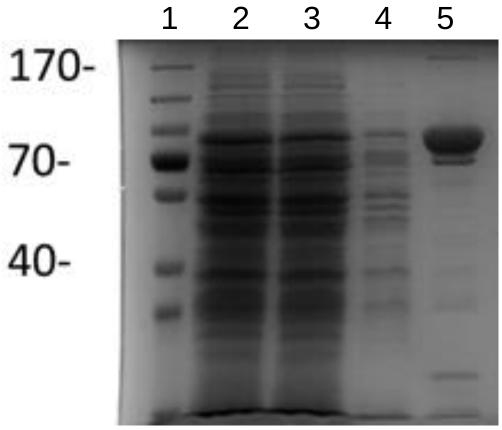

Figure D. Purification of CopB. Lane 1, molecular weight markers with 170, 70, and 40 kDa indicated to the left of the figure; lane 2, crude extract of induced cells; lane 3, Ni-NTA column flow-through; lane 4, column wash with 10 mM imidazole in JD-buffer; lane 5, elution of CopB with 200 mM imidazole in JD-buffer. The figure shows a 10% polyacrylamide/SDS gel stained with Coomassie blue.

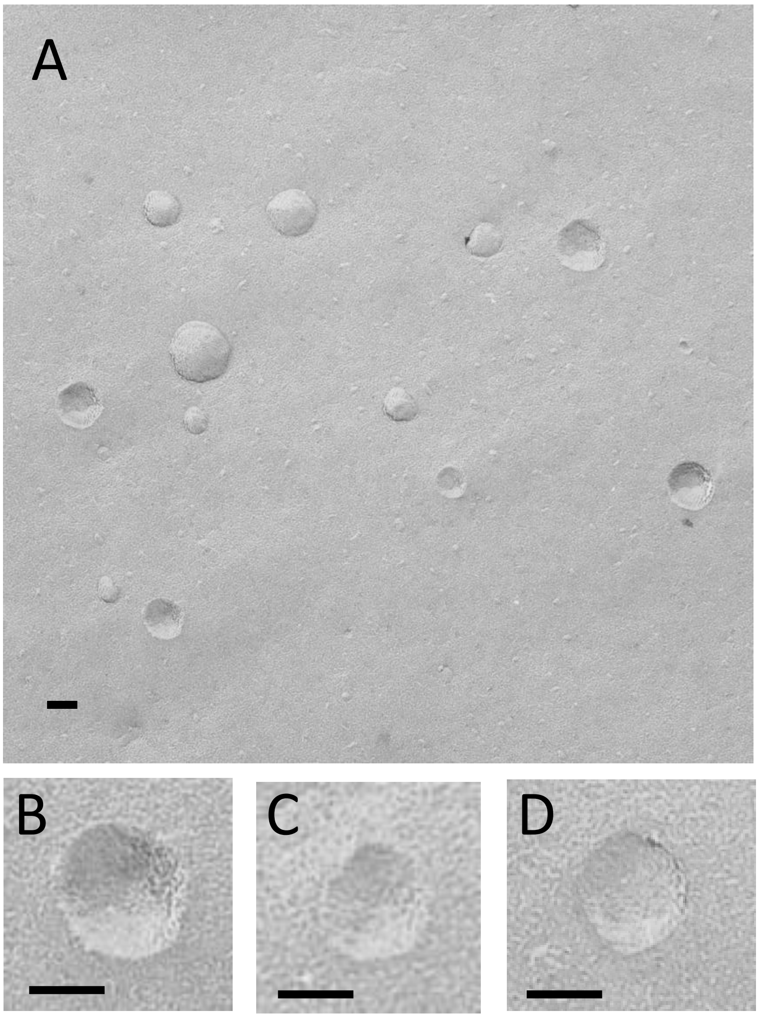

Figure E. Freeze-fracture electron micrographs of CopB proteoliposomes. (A), Low-magnification survey of proteoliposomes with a lipid/protein weight ratio of 50, prepared by octyl glucoside dialysis as outlined in the main text. (B), (C), and (D), concave fracture faces of individual vesicles, from the preparation shown in panel (A), at higher magnification to display particles. Shadowing was from the lower right and the bars correspond to 100 nm.

Supplementary methods

Freeze-fracture electron micrographs of proteoliposomes were prepared by concentrating the reconstituted vesicles 10-fold by exposing them in a dialysis tubing to dry polyethylene glycol 6000 for 30- 40 min. Less than 1 μl of sample was then frozen between copper strips in super-cooled liquid nitrogen and fractured under vacuum at -110 °C. Samples were etched for 1 min at this temperature, followed by cooling to - 260 °C for shadowing with 1 nm of platinum/carbon at an angle of 45° and 20 nm of carbon at 90°. The cleaned replicas were mounted on uncoated 400 mesh grids and observed in a Phillips 420 electron microscope at 100 kV.
